# A low-cost, portable, two-dimensional bioimpedance distribution estimation system based on the AD5933 impedance converter

**DOI:** 10.1016/j.ohx.2022.e00274

**Published:** 2022-02-08

**Authors:** Juan D. Muñoz, Víctor H. Mosquera, Carlos F. Rengifo

**Affiliations:** a Research Group of Automation, Universidad del Cauca, Colombia; b Department of Electronic Instrumentation and Control, Universidad del Cauca, Colombia

**Keywords:** Impedance distribution, Image reconstruction, Impedance measurement, Electrical bioimpedance

## Abstract

This study proposes a low-cost, portable, eight-channel electrical impedance tomograph based on the AD5933 impedance converter. The patterns for current injection and voltage measurement are managed by an Arduino Mega 2560 board and four 74HC4067 Texas Instruments multiplexers. Regarding the experimental results, the errors in the impedance estimates of an electrical circuit that represents a Cole model were less than 1.14% for the magnitude and 4.15% for the phase. Furthermore, the signal-to-noise ratio measured in a resistive phantom was 55.23 dB. Additional experiments consisted of placing five spheres of different size and conductivity in a saline tank, measuring their impedance through eight electrodes, and then generating impedance maps using the Electrical Impedance Tomography and Diffuse Optical Tomography Reconstruction Software (EIDORS). These maps were different for each sphere, suggesting the proposed prototype as a promising alternative for medical applications.

**Specifications table**

**Table:** 

Hardware name	System of estimation 2D of bioimpedance distribution (SE2DoBID*)*
Subject area	*Engineering and Material Science*
Hardware type	*Measurement bioimpedance system*
Open-source license	*CC BY 4.0*
Cost of hardware	*$80.5 USD*
Source File Repository	https://doi.org/10.17632/27m65n9mk7.1

## Hardware in context

Bioimpedance measurement (BIM) is a non-invasive, radiation-free technique for studying biological tissues [1]. BIM is based on the injection of an alternating current and the measurement of the resulting voltages [1]. The injection of current and the measurement of voltages is carried through an arrangement of electrodes located on the surface of the object under study. For BIM, there are two main approaches. The first uses Ohm's Law to relate the injected current and the measured voltages to provide information about the internal composition of biological tissues [2]. The second approach is electrical impedance tomography (EIT), which provides maps of the spatial distribution of electrical conductivity [1], [3], [4], [5].

BIM has been used successfully in the assessment of knee joint injury [1], respiratory monitoring [6], diagnosis of lung injury [2], [7], detection of cancerous tissue [8], pneumothorax [9], evaluation of soft tissue hydration [10], intracranial hemorrhage detection [11], bladder filling monitoring [12], body composition assessment [13], skeletal muscle mass estimation [14], evaluation of edema [15]. In addition, BIM and myographic signals have been combined to assess chronic obstructive pulmonary disease [16]. BIM has also been used to recognize wrist hand gestures [17], [18], [19], [20]. These applications show the potential of EIT in medical applications.

**Fig. 1:**
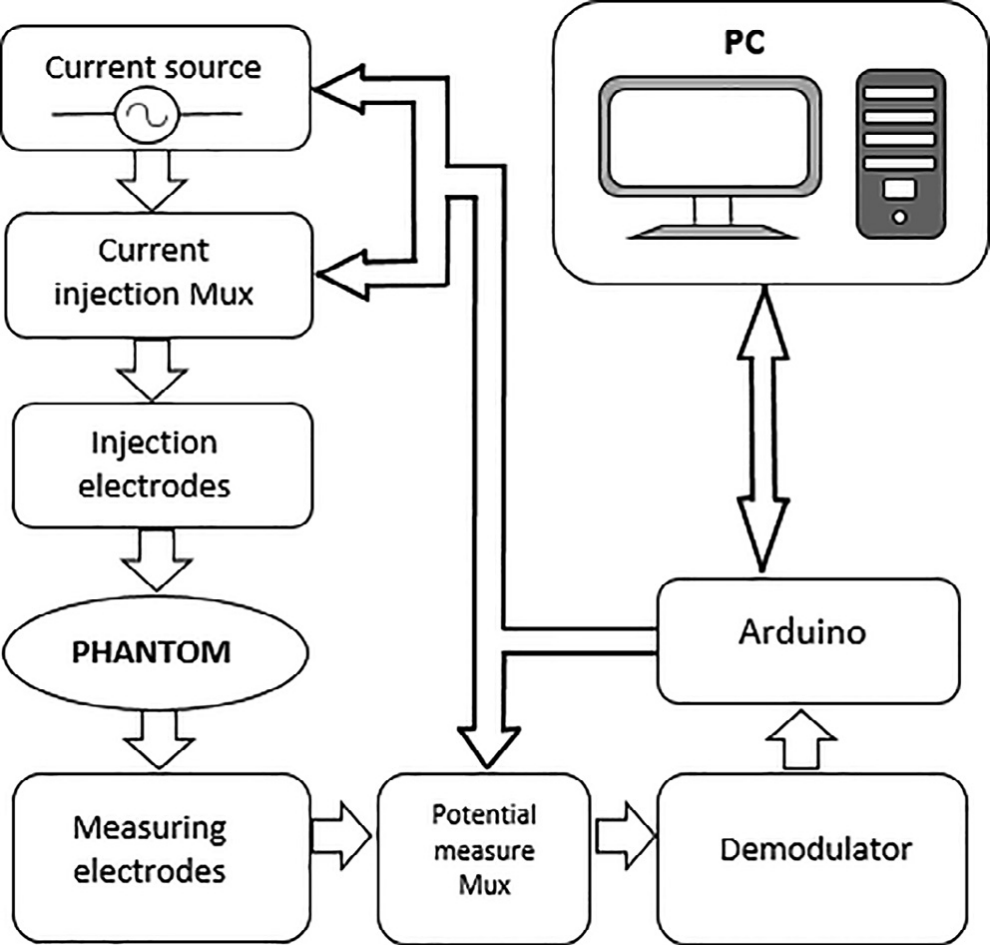
General scheme of a BIM system.

The hardware for EIT is evaluated in terms of spatial and temporal resolution. Spatial resolution depends on signal-to-noise ratio (SNR) and on the number of electrodes, while temporal resolution refers to the number of frames per second that the EIT device acquires [4]. Fig. 1 shows the hardware architecture for a two-dimensional (2D) EIT system [5], [21].

In the EIT literature, the authors use sinusoidal currents with different amplitudes and frequencies. For example, in [18], a 300 uA sinusoidal current at a frequency of 40 kHz was used; while in [17], [20], frequencies were 200 kHz and 40 kHz, respectively. In [22], [23], a 4 mA current signal with a frequency of 125 kHz was used. In [24], the amplitude of the sinusoidal current was 3 mA and the frequency took the values 50 kHz, 100 kHz, 250 kHz, 500 kHz and 1 MHz [25] shows that the BIM system based on the AD5933 achieves the best performance for a current of 1 mA at a frequency of 50 kHz. As a result, the present study, also based on the AD5933, will use these two parameters to generate impedance distribution images.

The proposed EIT system has design and sampling rate advantages compared to previously developed prototypes. In terms of design, the core of our BIM system is the AD5933 impedance converter, which includes a sinusoidal signal generator, a digital signal processor (DSP), and an analog-to-digital converter (ADC) on a single chip. In the devices presented in [26], [27], the DSP and the ADC are separated, which increases the sensitivity to noise and the probability of failure. Another advantage of our prototype is that, unlike the original configuration of the AD5933, it does not require an additional circuit to measure impedance less than 1 kΩ. Regarding sampling frequency, the proposed EIT system acquires 100 frames per second, being faster than the EIT prototype presented in [20].

## Hardware description

**Fig. 2:**
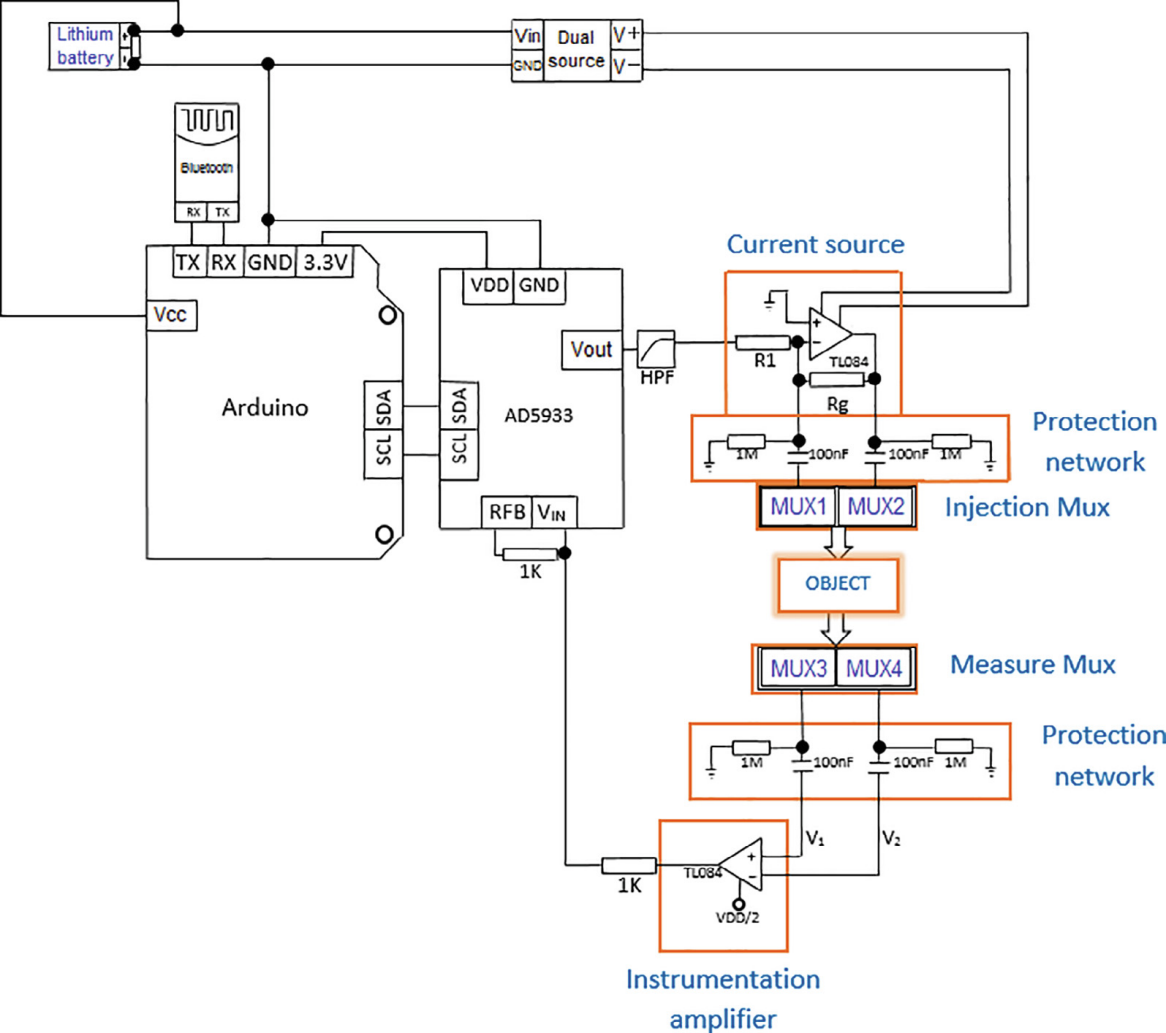
Scheme of the proposed EIT system.

The proposed EIT system comprises the following elements: i) one AD5933 impedance converter that generates an alternating voltage signal (V_out_), which passes through a high pass filter (HPF) to remove DC components; ii) a voltage-controlled current source that converts the voltage signal generated by the AD5933 into a 1-mA alternating current; iii) four Texas Instruments 74HC4067 multiplexers to generate injection and measurement patterns; iv) an instrumentation amplifier that measures voltage differences between pairs of electrodes; v) one DSP that calculates the real and imaginary parts of the load impedance; vi) one Arduino Mega 2560 board, which defines the frequency of the signal to be generated by the AD5933, manages the multiplexers, and transmits the estimated impedance to a personal computer through a Bluetooth module (Fig. 2). The EIT system includes a resistive-capacitive protection network connected in series to each multiplexer channel. This network filters the DC components of the current signals to comply with the IEC60601 safety standard for medical equipment [28].

### Impedance converter AD5933

The AD5933 is an impedance converter comprising a 27-bit resolution sinusoidal signal generator, an internal oscillator operating at 16 MHz maximum clock frequency (MCLK), a 12-bit ADC with a sampling rate of 1 megasample per second and a DSP. The frequency of the signal generator ranges from 1 to 100 kHz, with a resolution of 0.1 Hz. The AD5933 measures impedances from 1 kΩ to 10 MΩ [25], [29]. The AD5933 was selected for our prototype because impedances are estimated using Discrete Fourier Transform (DFT) rather than in-phase and quadrature demodulation, which is known to be sensitive to deviations from the sinusoidality assumption in the signal to be measured. The AD5933 applies a sinusoidal signal to the object under study and measures the resulting voltage across a shunt resistor. The real and imaginary parts of the first harmonic of this voltage are obtained through DFT. Hence, the high order harmonics, created by nonlinearities in the load impedance, are not considered for the impedance estimation.

#### Alternating signal generation and impedance measurement

The internal clock of the AD5933 is configured to work at the MCLK frequency. In addition, the peak-to-peak amplitude of V_out_ (Fig. 2) is configured to one volt by setting to one the bits 1 and 2 of the register 0x80 of the AD5933 (Table 1). The frequency of the signal V_out_ is programmed using Eq. (1).

(1) $$F_{S}=\left(\frac{F_{r}}{\frac{\mathrm{MCLK}}{4}}\right)\times 2^{27}$$

**Table 1:** Configuration of the control register 0x80 of the AD5933.

**Bit 7**	**Bit 6**	**Bit 5**	**Bit 4**	**Bit 4**	**Bit 2**	**Bit 1**	**Bit 0**
0	1	0	0	X	1	1	1
Frequency generation in repetition mode				This bit is not used	The peak-to-peak amplitude of $V_{\mathrm{OUT}}$		Gain of the PGA

To apply Eq. (1), both Fr and MCLK should be in Hz. For Fr = 50E3 Hz and MCLK = 16E6 Hz, the resulting decimal value for FS is 1677721, which is equivalent to the hexadecimal number 0 × 199999. The 8-bit numbers 0 × 19, 0 × 99, and 0 × 99 are written to the register addresses 0 × 82, 0 × 83, and 0 × 84, respectively. Since the proposed system operates at a single frequency of 50 kHz, it is necessary to activate the frequency repetition mode of the AD5933. This is done by assigning 0, 1, 0, and 0 to bits 7, 6, 5, and 4 of register 0 × 80 (Table 1). The AD5933 programmable gain amplifier (PGA) can be configured to multiply the ADC input signal by five or by one. In the latter case, bit 0 of register 0 × 80 is set to one, and otherwise, to zero. In our prototype, register 0 × 80 is configured, as indicated in Table 1.

The DSP applies the DFT to the ADC input, generating the real (R) and imaginary (I) parts of the measured impedance [29]. The real part is stored in registers 0 × 94 and 0 × 95, whereas the imaginary part is stored in registers 0 × 96 and 0 × 97 [29]. This study proposes an analog front end, which is included in our prototype to solve the two main limitations of the AD5933: (i) the AD5933 measures impedance ranging from 1 kΩ to 10 MΩ [25], [29], but most biological tissues are below 1 kΩ [30], and (ii) the AD5933 uses voltages instead of currents, which is considered potentially dangerous in medical applications as the resulting current may be greater than the limits defined by IEC60601 [31].

### Analog front end

**Fig. 3:**
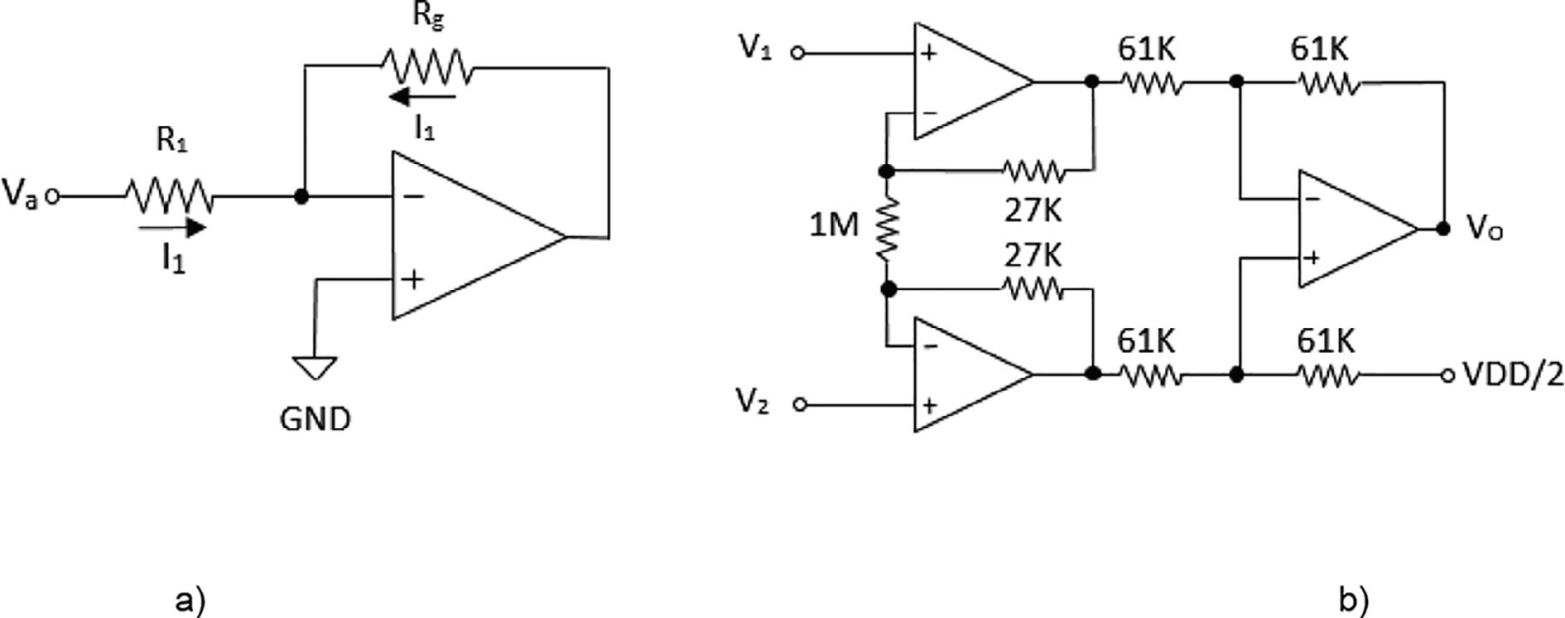
a) Voltage-to-current converter; b) Instrumentation amplifier.

**Fig. 4:**
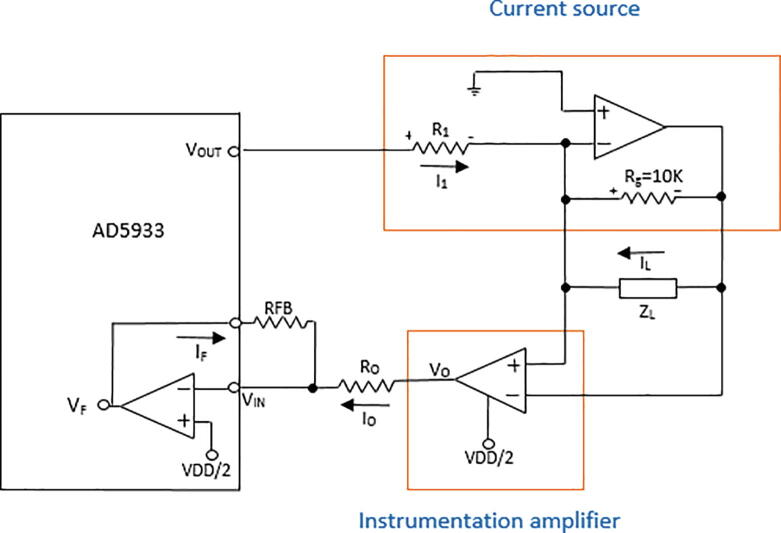
Simplified diagram of an electrical impedance tomography (EIT) system.

The proposed analog front-end for the AD5933 comprises a high-pass filter (HPF) (Fig. 2), a voltage-to-current converter (VCCS) (Fig. 3a), and an instrumentation amplifier (Fig. 3b). The HPF is a first order system composed by a 100 kΩ resistor and a 10 nF capacitor to remove the DC components of V_out_. The HPF output is connected to VCCS [25], which consists of a TL084 operational amplifier and two resistors (R_1_ = 1 kΩ; R_g_ = 10 kΩ). The integrated TL084 is chosen for its fast response, low power consumption and low sensitivity to noise [32]. Because the impedance is supposed to vary from tens to hundreds of ohms [30], R_g_ = 10 kΩ ensures that current I_1_ (Fig. 4) flows mainly through Z_L_, which is connected in parallel to R_g_ (Fig. 4). The unity gain instrumentation amplifier is implemented by a TL084, which receives, through its non-inverting input, the voltages on the electrodes connected to Z_L_. Since the ADC converter of the AD5933 is unipolar, a potential (VDD/2) is added to the voltage V_0_ (Fig. 4). The DSP of the AD5933 calculates the real and imaginary parts of Z_L_, which are read by an Arduino Mega 2560 board through an inter-integrated circuit (I2C) protocol and sent to a personal computer using a Bluetooth module HC-05. The Arduino Mega 2560 board was selected because its high data acquisition rate and because it has enough digital pins to manage the four 74HC4067 multiplexers.

### Impedance estimation by EIT system

Fig. 4 shows a simplified diagram of the proposed EIT system.

When $Z_{L}$ is significantly less than $R_{g}=10$ kΩ, $I_{1}=$ ${-I}_{L}$. In such a case, voltages across $Z_{L}$ and $V_{o}$ are given by Eqs. (2), (3), respectively.

(2) $$V_{L}=-I_{1}*Z_{L}=-\frac{V_{\mathrm{OUT}}}{R_{1}}*Z_{L}$$

(3) $$V_{o}=V_{L}+0.5V_{\mathrm{DD}}$$

The currents $I_{o}$ and $I_{F}$ are obtained by applying Ohḿs law to the resistors $R_{o}$ and RFB:

(4) $$I_{o}=\frac{V_{o}-0.5V_{\mathrm{DD}}}{R_{o}}$$

(5) $$I_{F}=\frac{V_{F}-0.5V_{\mathrm{DD}}}{\mathrm{RFB}}$$

The equation for $I_{o}$ is rewritten by replacing Eqs. (2), (3) in Eq. (4):

(6) $$I_{o}=-\frac{Z_{L}*V_{\mathrm{OUT}}}{R_{o}*R_{1}}$$

The voltage $V_{F}$, read by the AD5933, can be related to the load impedance $Z_{L}$ by knowing that $I_{o}=-I_{F}$. Hence, from Eqs. (5), (6) and considering that RFB=1 kΩ and $R_{o}=1$ kΩ

(7) $$-\frac{Z_{L}*V_{\mathrm{OUT}}}{R_{1}}={0.5V}_{\mathrm{DD}}-V_{F}$$

Solving for $Z_{L}$

(8) $$Z_{L}=\left(\frac{R_{1}}{V_{\mathrm{OUT}}}\right)V_{F}-\left(\frac{{{0.5V}_{\mathrm{DD}}*R}_{1}}{V_{\mathrm{OUT}}}\right)$$

When the AD5933 is used without the front-end described in Fig. 4, the impedance is estimated as follows [29]:

(9) $$\mathrm{Impedance}=\frac{1}{\mathrm{FG}∙mag}$$

where $\mathrm{mag}=\sqrt{R^{2}+I^{2}}$. The R and I integers are the values of the registers that contain the real and imaginary parts of the first harmonic of the voltage V_F_ (Fig. 4). FG is a gain factor calculated from a test performed on a $Z_{L}$ of known value [29]. A limitation of the AD5933 in its original configuration is that when the load is less than 1 kΩ, it requires an additional buffer circuit [29]. On the contrary, the proposed prototype does not suffer from this limitation and, therefore, Eq. (8) is also valid for low impedances. Another advantage of our prototype is that the estimated impedance is proportional to V_F_ (Eq. (8)), while in Eq. (9) this relationship is nonlinear.

### Injection and measurement patterns

**Fig. 5:**
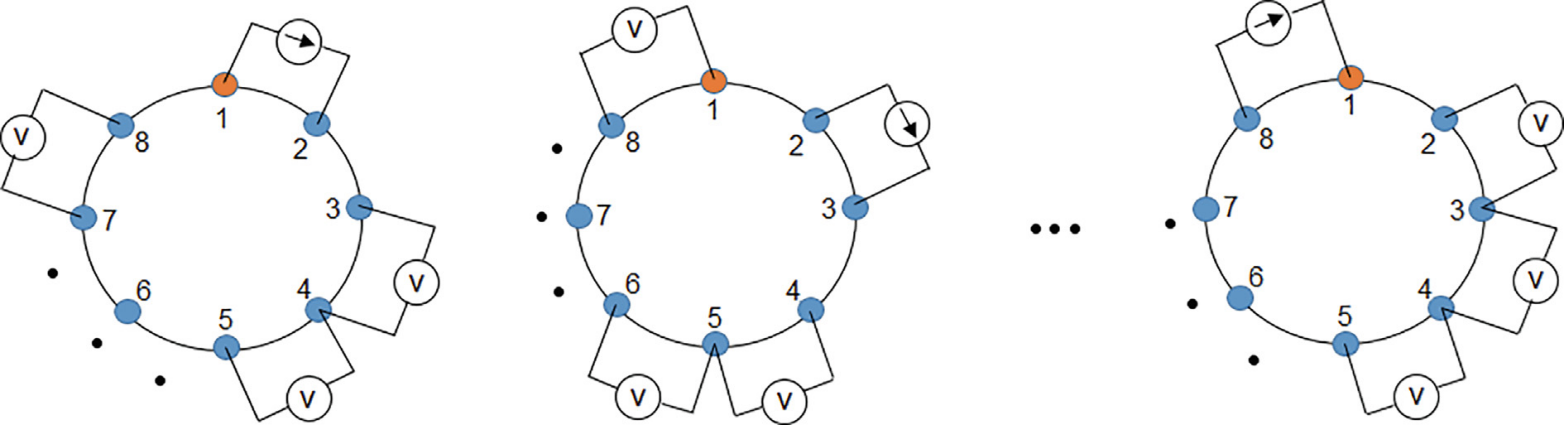
Injection and measurement pattern.

**Fig. 6:**
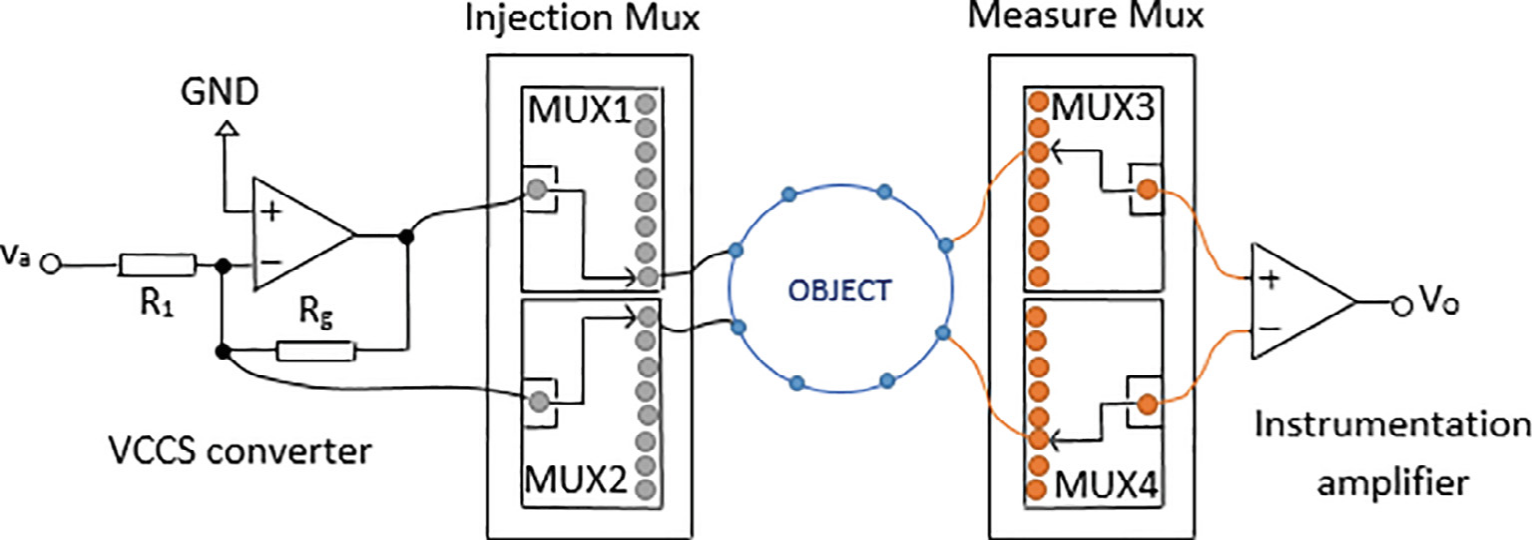
Connection of multiplexing module.

Current injection and voltage measurement is done using an adjacent pattern, which is based on the four-electrode method, which basically consists of injecting current through one pair of electrodes and measuring the voltage through another pair of electrodes [33]. The use of four 74HC4067 multiplexers is essential since they allow selecting the pairs of current injection electrodes and voltage measurement until completing the adjacent pattern presented in Fig. 5 [33]. The 74HC4067 multiplexer was chosen for its high speed switching and low sensitivity to noise [34]. An Arduino Mega 2560 board manages the selection of the channels of each multiplexer. Fig. 6 shows the connection between the multiplexers, the VCCS converter and the instrumentation amplifier.

### Polarization of the EIT system

**Fig. 7:**
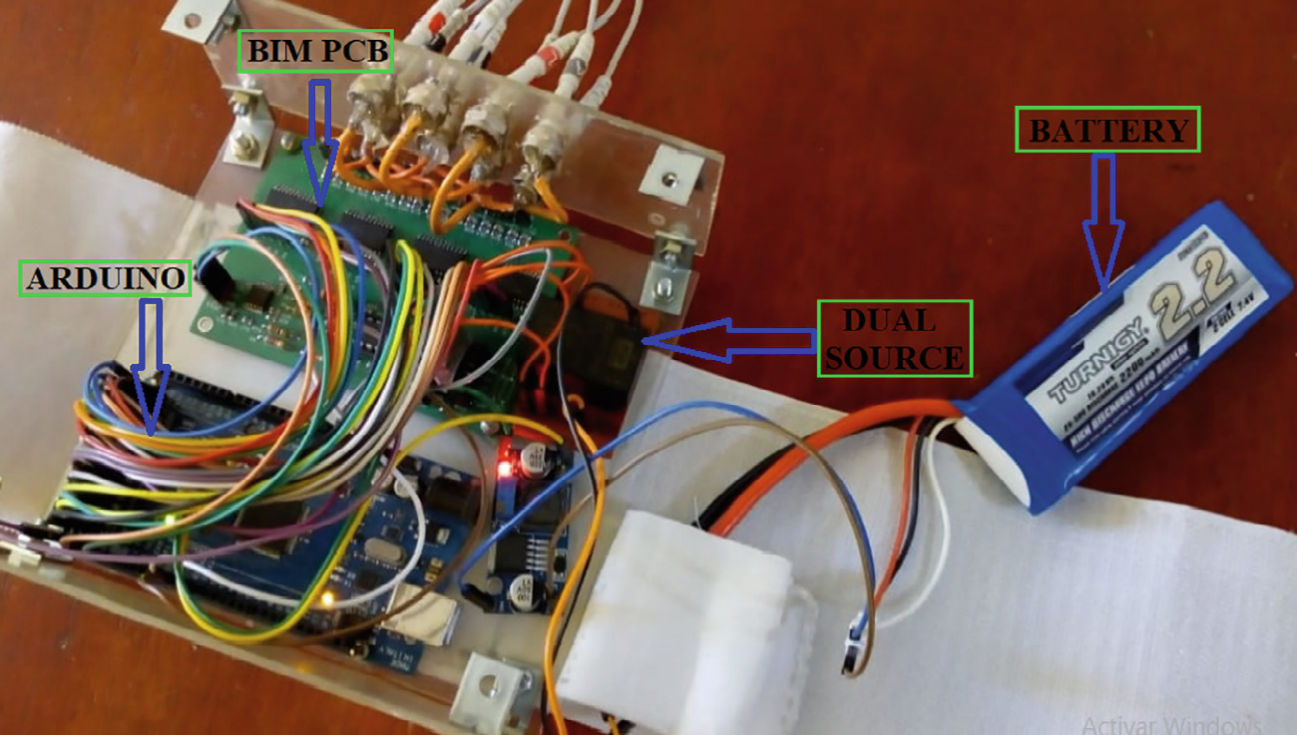
BIM system polarization*.*

The AD5933 impedance converter and the HC-05 Bluetooth module must be powered at 3.3 V and 5 V, respectively, through the Arduino Mega 2560 board; similarly, the SCL and SDA ports of the AD5933 are biased to 3.3 V using a pair of 10 kΩ resistors. Subsequently, a 7.4 V lithium battery is connected to the Arduino and to a DCWN03E-05 dual source module. Dual source energize to the multiplexers, the VCCS and the instrumentation amplifier (Fig. 7).

**Fig. 8:**
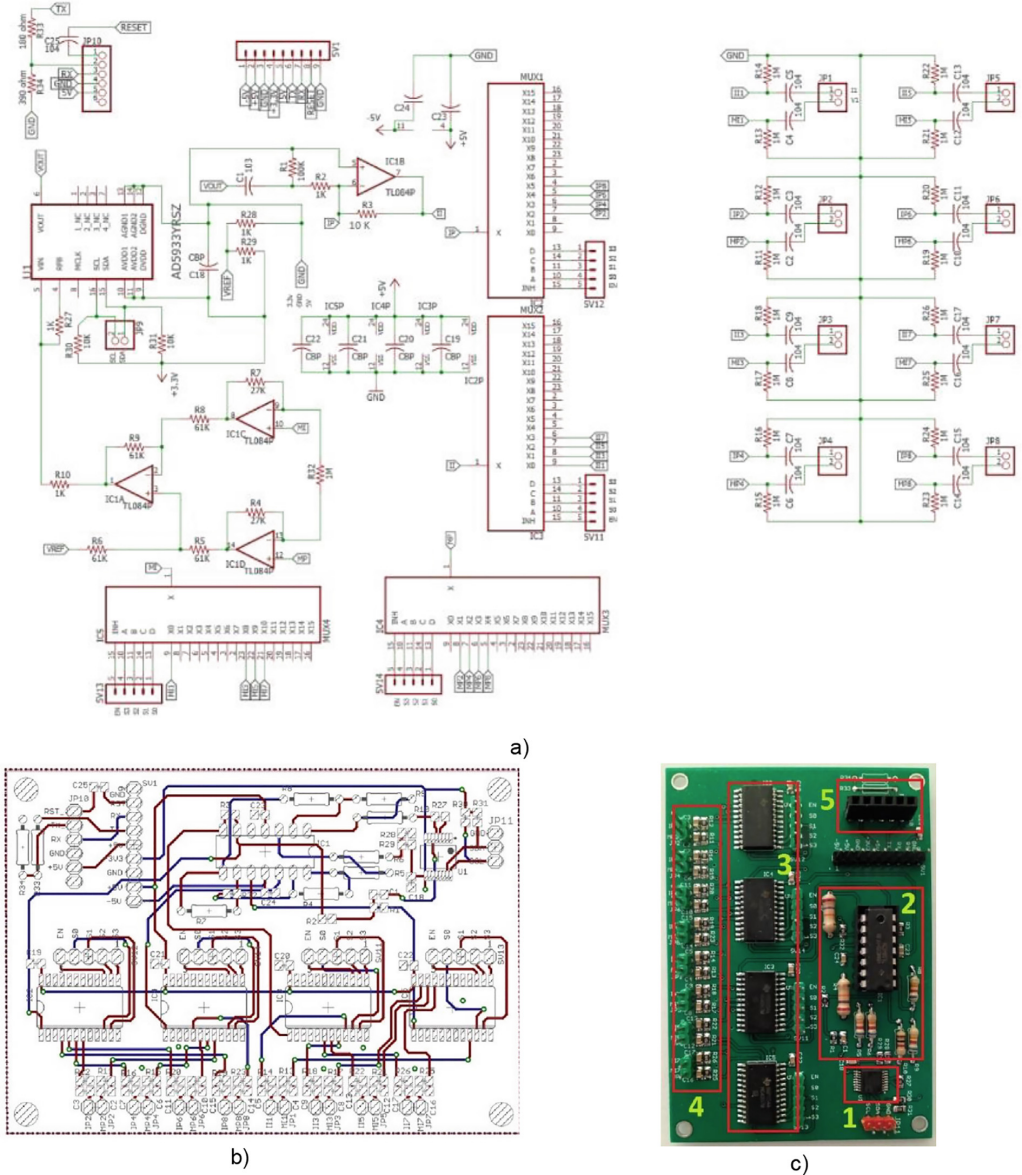
a) BIM system diagram in Eagle, b) BIM system PCB in Eagle, c) BIM system (1: AD5933; 2: Current source + Amp. Instrumentation; 3: Multiplexers; 4: Protection network + Electrodes; 5: Bluetooth).

**Fig. 9:**
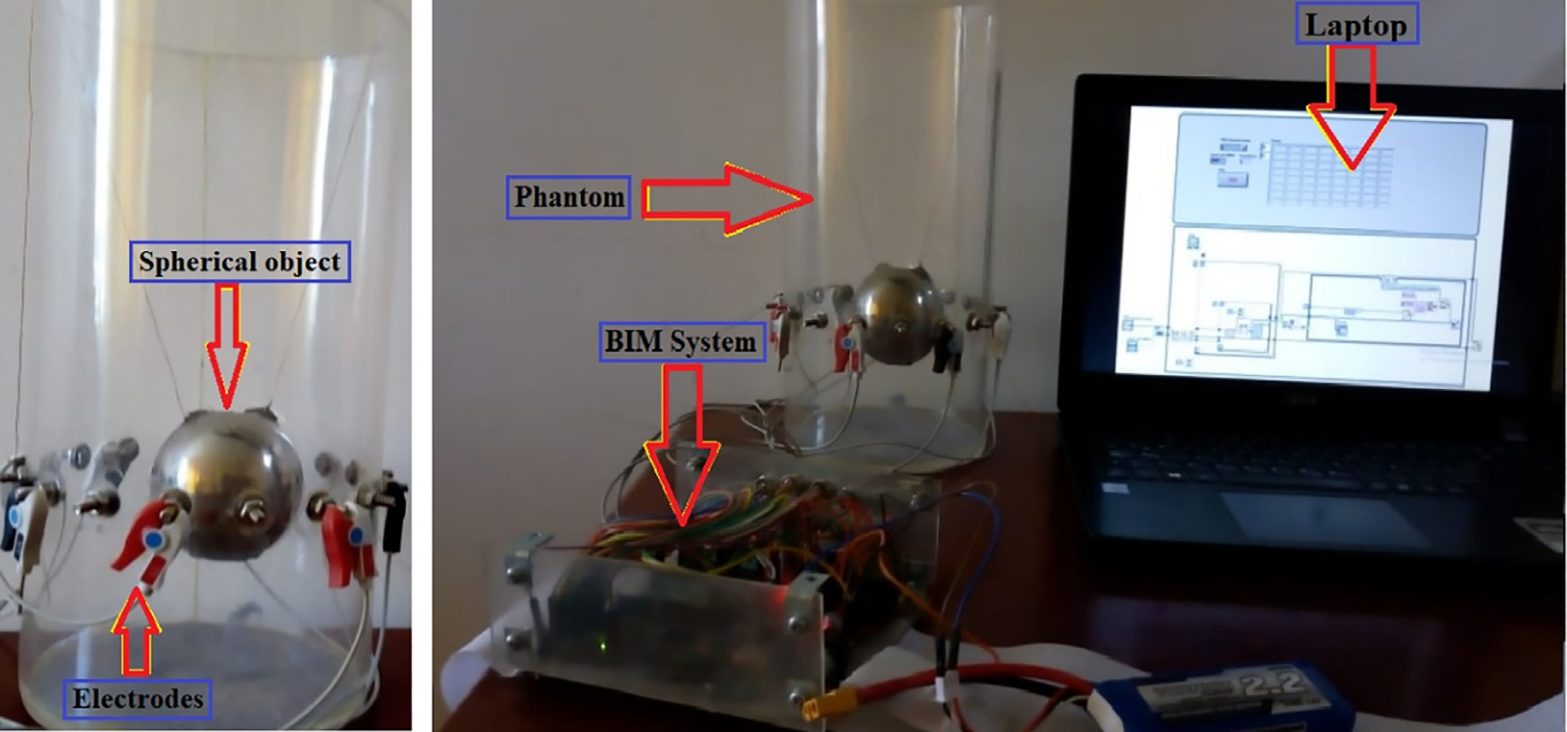
Impedance measurement.

### Wireless communication and data acquisition

In biomedical applications, the object under study should be at a distance greater than 1 m from computers to avoid Radio-Frequency interference. This recommendation can be achieved through the use of wired or wireless data transmission between the data collecting device and the computer used to process information. In the present prototype, the communication between the Arduino board and the computer could have been implemented through Universal Serial Bus communication, which is simpler and more reliable than the wireless option. Nevertheless, the proposed prototype uses Bluetooth communication to guarantee complete electrical isolation from the power grid (Fig. 2). On the other hand, the connection between the electrodes and the Arduino board is wired as it is the case of most bioimpedance measurement prototypes [1], [2], [3], [5], and [35].

The computer used for the experiments described below runs a LabVIEW application that stores the data sent by the Arduino board in text files. These text files are post-processed using EIDORS, which is open source and implements various image reconstruction algorithms. In future prototypes, a Secure Digital (SD) memory card will be added to the Arduino board to prevent data loss in the event of a wireless communication failure. A bioimpedance measurement system using an SD card for data storage is described in [36].

## Design files

The following table shows the figures that correspond to the design of the proposed BIM system.

**Table:** 

Design file name	File type	Open source license	Location of the file
*General scheme of a BIM system*	*Figure (PNG)*	*CC BY 4.0*	*Included in the article (*Fig. 1*)*
*Scheme of the proposed BIM system*	*Figure (PNG)*	*CC BY 4.0*	*Included in the article (*Fig. 2*)*
*Voltage-to-current converter*	*Figure (PNG)*	*CC BY 4.0*	*Included in the article (*Fig. 3*a)*
*Instrumentation amplifier*	*Figure (PNG)*	*CC BY 4.0*	*Included in the article (*Fig. 3*b)*
*Simplified diagram of an electrical impedance tomography (EIT) system*	*Figure (PNG)*	*CC BY 4.0*	*Included in the article (*Fig. 4*)*
*Injection and measurement pattern*	*Figure (PNG)*	*CC BY 4.0*	*Included in the article (*Fig. 5*)*
*Connection of multiplexing module*	*Figure (PNG)*	*CC BY 4.0*	*Included in the article (*Fig. 6*)*
*BIM system polarization*	*Figure (PNG)*	*CC BY 4.0*	*Included in the article (*Fig. 7*)*
*BIM system diagram in Eagle*	*Figure (PNG)*	*CC BY 4.0*	*Included in the article (*Fig. 8*a)*
*BIM system PCB in Eagle*	*Figure (PNG)*	*CC BY 4.0*	*Included in the article (*Fig. 8*b)*
*BIM system*	*Figure (PNG)*	*CC BY 4.0*	*Included in the article (*Fig. 8*c)*
*Impedance measurement*	*Figure (PNG)*	*CC BY 4.0*	*Included in the article (*Fig. 9*)*

Files corresponding to the design and programming of the BIM system are in the System BIM folder of the repository https://doi.org/10.17632/27m65n9mk7.1

• **BIM_System_Design:** This folder contains files corresponding to the design of the BIM system and their respective modules.
• **Matlab_Script:** This folder contains files corresponding to Matlab scripts used for calculating the SNR (file SNR.m) and global impedance (file impedance_measurement.m).
• **Octave_Files:** The Octave script that contains the (EIDORS) algorithm used for reconstructing impedance images (eidors.m).
• **PCB_files:** This folder contains the EAGLE design files of the PCB (pcb2.BRD and pcb2.SCH files).
• **Arduino_Files:** This folder contains the Arduino sketch for operating the BIM system (file BIM_AD5933.ino).
• **LabVIEW_Files:** This contains the LabVIEW application for storing impedance measurements (file BIM_AD5933.vi).

## Bill of materials

The list of materials used in the design of the BIM system are presented in the following table.

**Table:** 

Designator	Component	Number	Cost per unit -currency	Total cost -currency	Source of materials	Material type
*Impedance converter*	*AD5933, 1MSPS, 12 bits*	*1*	*$35.96 USD*	*$35.96 USD*	digikey	*Other*
*Operational amplifier*	*TL084 JFET-Input*	*1*	*$0.64 USD*	*$0.64 USD*	sigmaelectronica	*Other*
*Multiplexer*	*74HC4067, High-Speed CMOS Logic 16 Channel Analog Multiplexer-Demultiplexer*	*4*	*$2.68 USD*	*$10.72 USD*	mercadolibre	*Other*
*Arduino*	*Arduino Mega 2560, 16 MHz crystal oscillator*	*1*	*$12.07 USD*	*$12.07 USD*	electrotekmega	*Other*
*Bluetooth*	*Bluetooth HC-05 v2.0*	*1*	*$4.83 USD*	*$4.83 USD*	electrotekmega	*Other*
*Resistor*	*1 KΩ, ¼ w*	*6*	*$0.0089 USD*	*$0.054 USD*	mercadolibre	*Other*
*Resistor*	*10 KΩ, ¼ w*	*2*	*$0.0089 USD*	*$0.017 USD*	mercadolibre	*Other*
*Resistor*	*100 KΩ, ¼ w*	*1*	*$0.0089 USD*	*$0.0089 USD*	mercadolibre	*Other*
*Resistor*	*27 KΩ, ¼ w*	*2*	*$0.0089 USD*	*$0.017 USD*	mercadolibre	*Other*
*Resistor*	*61 KΩ, ¼ w*	*4*	*$0.0089 USD*	*$0.035 USD*	mercadolibre	*Other*
*Resistor*	*1 MΩ, ¼ w*	*17*	*$0.0089 USD*	*$0.15 USD*	mercadolibre	*Other*
*Capacitor*	*10 nF*	*1*	*$0.027 USD*	*$0.027 USD*	mercadolibre	*Ceramic*
*Capacitor*	*100 nF*	*16*	*$0.027 USD*	*$0.43 USD*	mercadolibre	*Ceramic*
*Lithium Battery*	*LiPo Turnigy Battery Nano-Tech 7.4 V (2S) 300 mAh 35-70C*	*1*	*$9.12 USD*	*$9.12 USD*	demosspro	*Compound*

## Build instructions

The correct operation of the BIM system requires the following operations before using the device (Fig. 7):

• With 3.3 V (VDD), polarize the impedance converter AD5933 and its ports SCL-SDA (I2C interface) using the Arduino Mega 2560 board.
• Polarize the HC-05 Bluetooth module with 5 V via Arduino Mega 2560 board for wireless communication.
• Power the dual-source module with a 7.4-V Li-Po battery.
• Energize the current source, instrumentation amplifier, and multiplexers with ±5 V, supplied by the dual-source module. In this step, it is recommended to identify the positive and negative voltages of the system to make a correct connection of the modules, thereby avoiding irreversible damage to the BIM prototype.

Fig. 8 shows the circuit diagram, the PCB layout, and the BIM system implementation.

## Operation instructions

Once the BIM system has been configured and energized (Section 5), measurements are obtained by following the procedure described below (Fig. 9):

• Connect the electrodes around the object under study.
• Load the file located in the *Arduino_Files* folder to the Arduino board.
• Execute the LabVIEW application for the acquisition and storage of the impedance measurements. This software is included in the *LabVIEW_Files* folder.
• Execute the Matlab script located in the *Matlab_Script* folder to calculate the global impedance index.
• Execute the Octave script located in the *Octave_Files* folder to obtain the impedance distribution using EIDORS.

After the measurements are completed, the 7.4 V lithium battery is disconnected and then the electrodes attached around the object under study are removed.

## Validation and characterization

### BIM system calibration

**Fig. 10:**
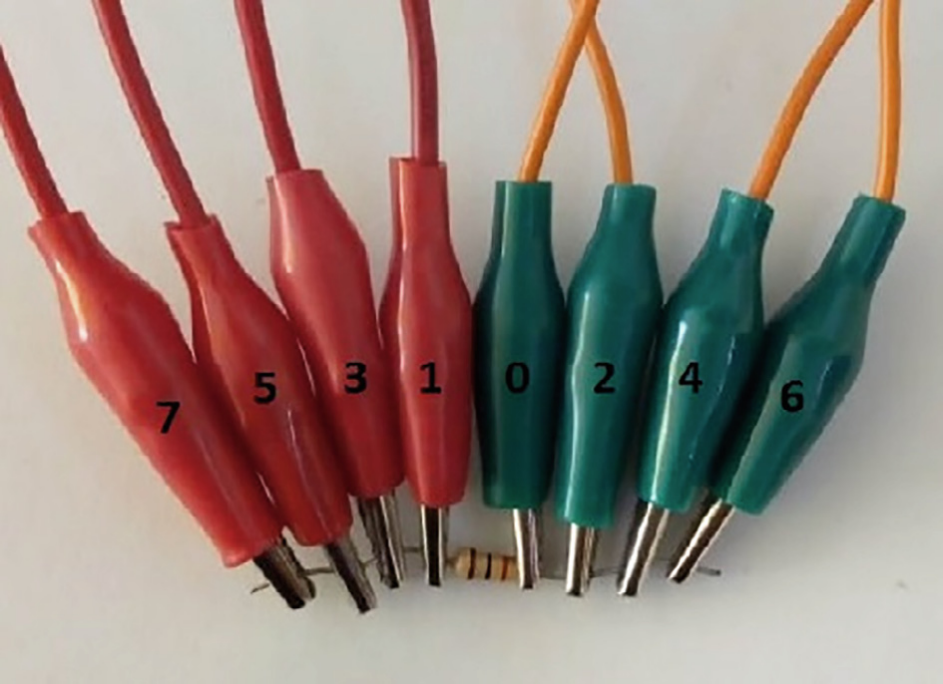
Electrode arrangement for calibration.

The AD5933 is calibrated using the procedure proposed by the manufacturer [29]. The first step is to connect ports 0, 2, 4, and 6 to one terminal of a 986-Ω resistance and ports 1, 3, 5, and 7 to the other terminal (Fig. 10). Subsequently, average 50 measurements. In our experiments, the average impedance was 992 Ω, presenting a relative error of 0.8%.

### Signal-to-noise ratio

**Fig. 11:**
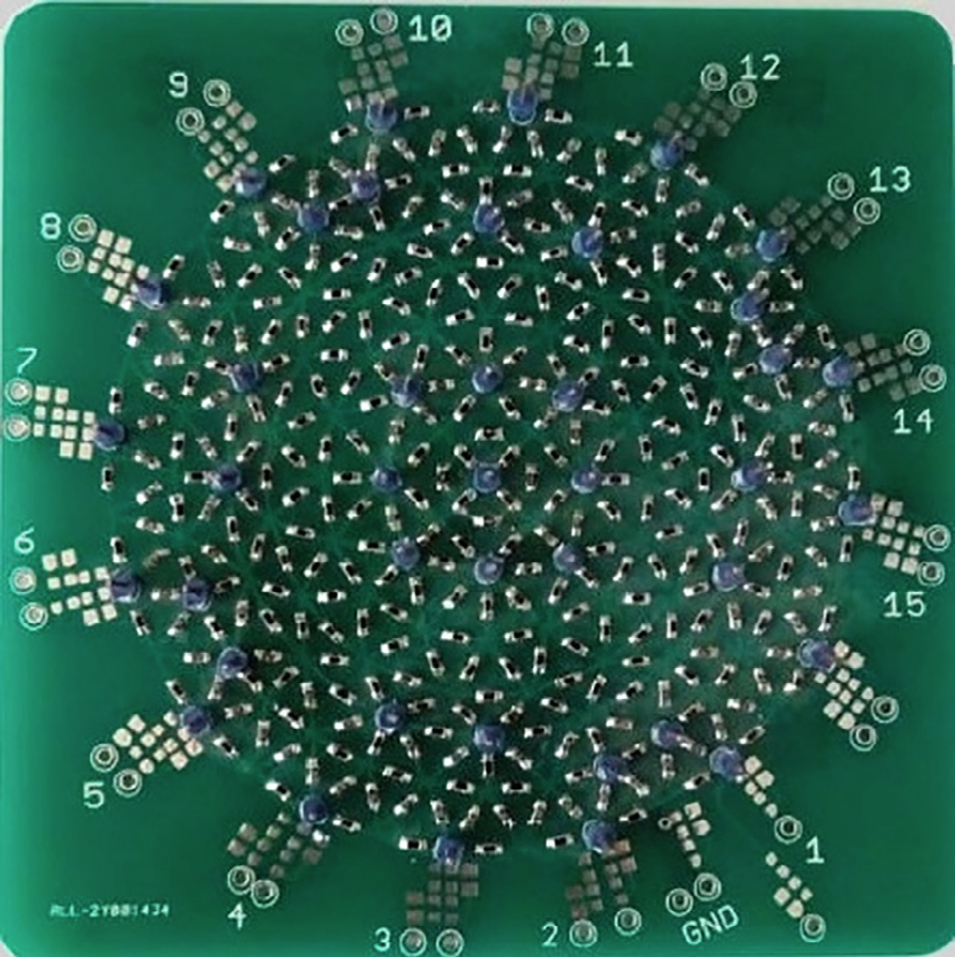
Resistive phantom.

Resistive phantoms (Fig. 11) are used to evaluate the performance of BIM systems in terms of SNR and precision. This is because they provide predictable, stable and reproducible signals [37], [38]. These phantoms are interconnections of resistances of known value that form a specific topology and have the ability to generate localized conductivity disturbances [37].

In the present work, current is initially injected through electrodes 1 and 2, and voltages are measured at the following five pairs of electrodes: (3,4), (4,5), (5,6), (6,7), and (7,8). Since there are eight possibilities to select the electrode 1, a frame is a set of 40 measurements (Fig. 5). The sampling rate, measured in frames per second, indicates the number of frames obtained in one second by the BIM system. The SNR was calculated using 50 measurements obtained from a resistive phantom (Fig. 11) using Eq. (10) [37]. In such an equation, ${\overset{-}{m}}_{i}$ and $\sigma \left(m_{i}\right)$ represent the mean and standard deviation of the impedance between each pair of electrodes, respectively. The number of frames per second were modified to determine the best SNR. Table 2 shows the results of this experiment.

(10) $$\mathrm{SNR}=20\log\left(\frac{{\overset{-}{m}}_{i}}{\sigma \left(m_{i}\right)}\right)$$

**Table 2:** SNR on a resistive phantom.

Frames per second	SNR (dB)		
	Min	Max	Mean
**1000**	12.08	26.31	16.90
**200**	23.01	37.53	27.70
**100**	50.90	59.97	**55.23**
**50**	28.31	52.47	38.12
**33**	28.56	52.62	38.18
**25**	28.08	51.13	37.92
**20**	28.41	52.71	38.18

According to Table 2, the highest SNR (55.23 dB) is obtained at a sample rate of 100 frames per second. Therefore, to obtain the best contrast in the impedance distribution maps, this frequency will be used in the experiments presented in the subsequent sections. In future studies, electrodes with low noise sensitivity [39] will be used to increase the SNR above 55.23 dB.

### Phase and magnitude measurement on Cole’s model

**Fig. 12:**
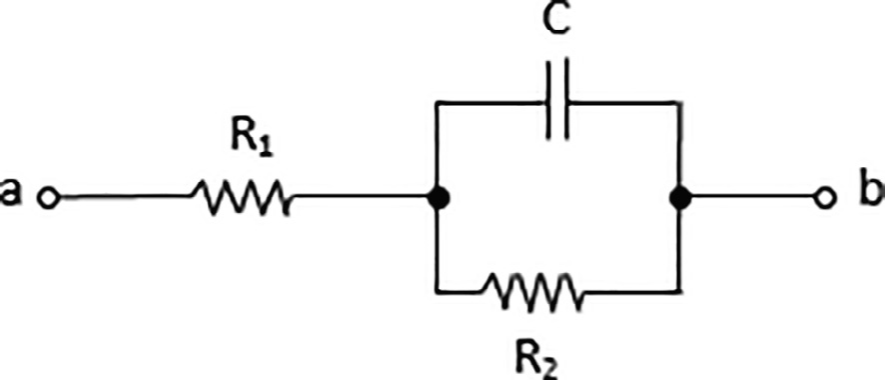
Cole’s model.

Cole's simple dispersion model (Fig. 12) comprises two resistors and a fractional order capacitor [40]. These three elements represent the conductivity and permittivity of a biological tissue [41], [42], [43]. In this work, the BIM prototype was validated by comparing the theoretical and estimated magnitude and phase of four Cole circuits. For all of them, R_2_ and C were set to 196 Ω and 100 nF, respectively, while R_1_ was different for each circuit (146 Ω, 461 Ω, 909 Ω, and 986 Ω). R_2_ and C were selected to keep the total impedance below 1 kΩ at the operating frequency of the BIM prototype (50 kHz).

In Table 3 and Table 4, the percentage of error is defined as the difference between the theoretical and estimated values of a variable, divided by its theoretical value. For each Cole circuit, the magnitude and phase were measured 50 times. The interquartile range for each data set was calculated as the difference between the thresholds that define the quartils three (Q3) and one (Q1) (Eq. (11)). Q3 is the lowest value that exceeds 75% of the items in a data set, and Q1 is the lowest value that exceeds 25% of those items.

(11) RQ=Q3-Q1

**Table 3:** Theoretical and measured magnitudes of Cole’s model at 50 kHz by averaging 50 frames.

R1 (Ω)	Theoretical Magnitude (Ω) of Cole’s circuit	Measured magnitude (Ω)		Relative error (%)
		Mean	Interquartile range	
146	154	155	5.5	0.65
461	467	462	58.2	1.07
909	914	912	13.4	0.22
986	991	977	34.7	1.41

**Table 4:** Theoretical and measured phase of Cole’s model at 50 kHz by averaging 50 frames.

R1 (Ω)	Theoretical Phase (°) of Cole’s circuit (degrees)	Measured phase (°)		Relative error (%)
		Mean	Interquartile range	
146	−11.60	−11.52	0.29	0.75
461	−3.81	−3.78	0.17	0.67
909	−1.94	−1.95	0.11	0.25
986	−1.79	−1.72	0.34	4.10

On the other hand, the percentage relative error is calculated by Eq. (12);

(12) $$E_{r}=\frac{I_{r}-I_{m}}{I_{r}}*100$$

where I_r_ is the actual or theoretical value and I_m_ the measured value, impedance and phase.

Table 3 and Table 4 show that the percentage errors for magnitudes and phases are lower than 2% and 5%, respectively. The small interquartile ranges in both tables indicate low noise sensitivity.

### Image reconstruction of impedance distribution

In general, phantoms are designed with materials that replicate the electrical properties of biological tissues [44], [45], [46]. The simplest phatoms are saline tanks, in which conductive and nonconductive objects are placed to test EIT prototypes [47], [48]. In this work, experiments were performed using a cylindrical tank of 7.5 cm in radius and 30 cm in height filled with saline solution (4 gr/L), three steel spheres with diameters of 15.7, 19.8, and 21.5 cm, and two plastic spheres with diameters of 12.5 and 25.8 cm. These five spheres were introduced one by one in the tank, and then an impedance distribution image was obtained for each sphere using EIDORS. In EIDORS, the impedance maps were obtained by applying the Gauss-Newton algorithm to a finite element model with 1600 nodes. The Noser algorithm was used as a regularization method to improve the quality of the maps.

**Fig. 13:**
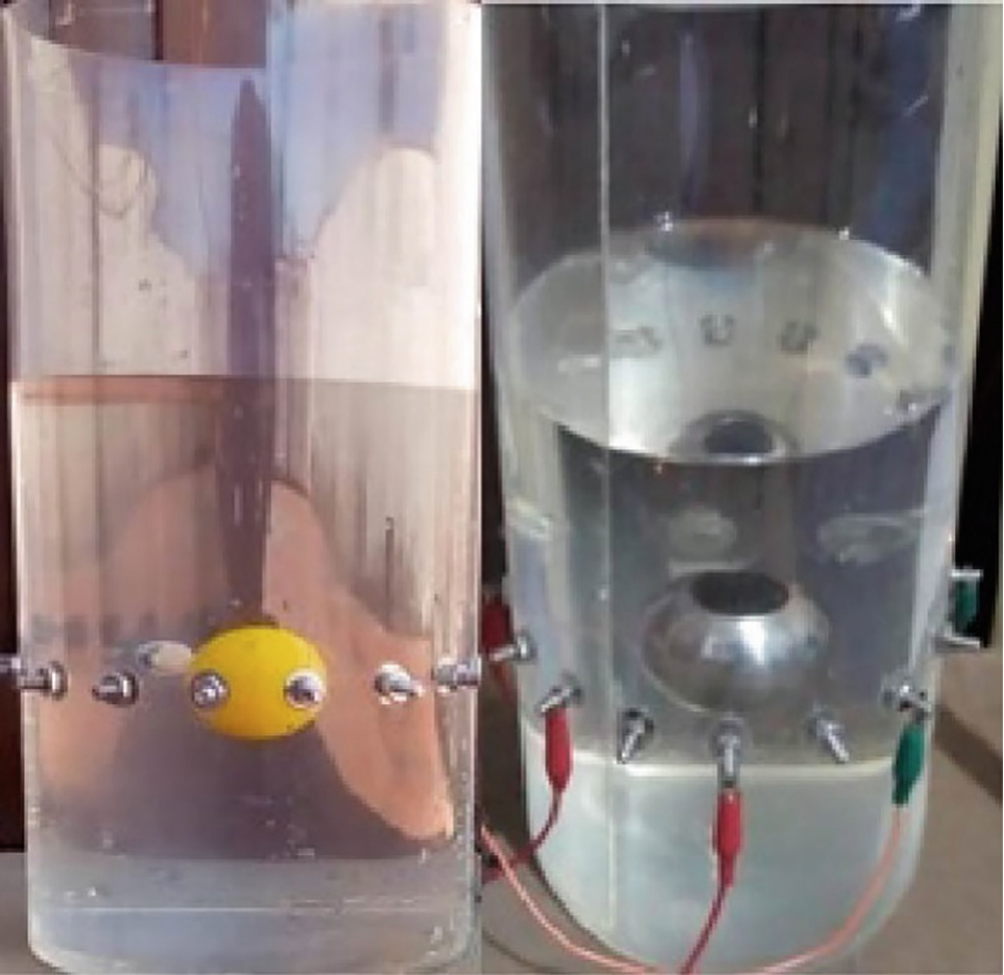
Location of spheres in a saline tank to obtain impedance distribution maps. The yellow sphere is nonconducting, whereas the gray sphere is made of stainless steel. (For interpretation of the references to colour in this figure legend, the reader is referred to the web version of this article.)

The saline tank with no spheres was used to obtain the homogeneous measurements. Subsequently, the spheres were placed one at a time in the tank, to obtain the non-homogeneous measurements. The homogeneous and non-homogeneous measurements are used to determine the ability of the BIM system to detect variations in the impedance and size of the spheres. Fig. 13 depicts the location of the spheres in the saline tank.

**Fig. 14:**
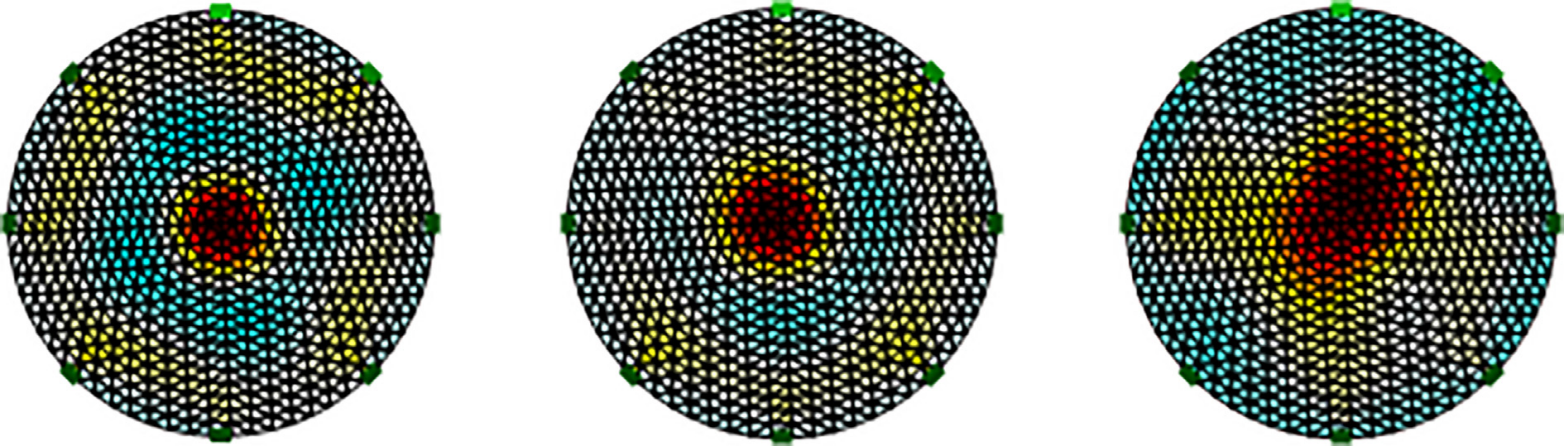
EIT image reconstruction of steel spheres: small on the left, median in the center, and big on the right.

**Fig. 15:**
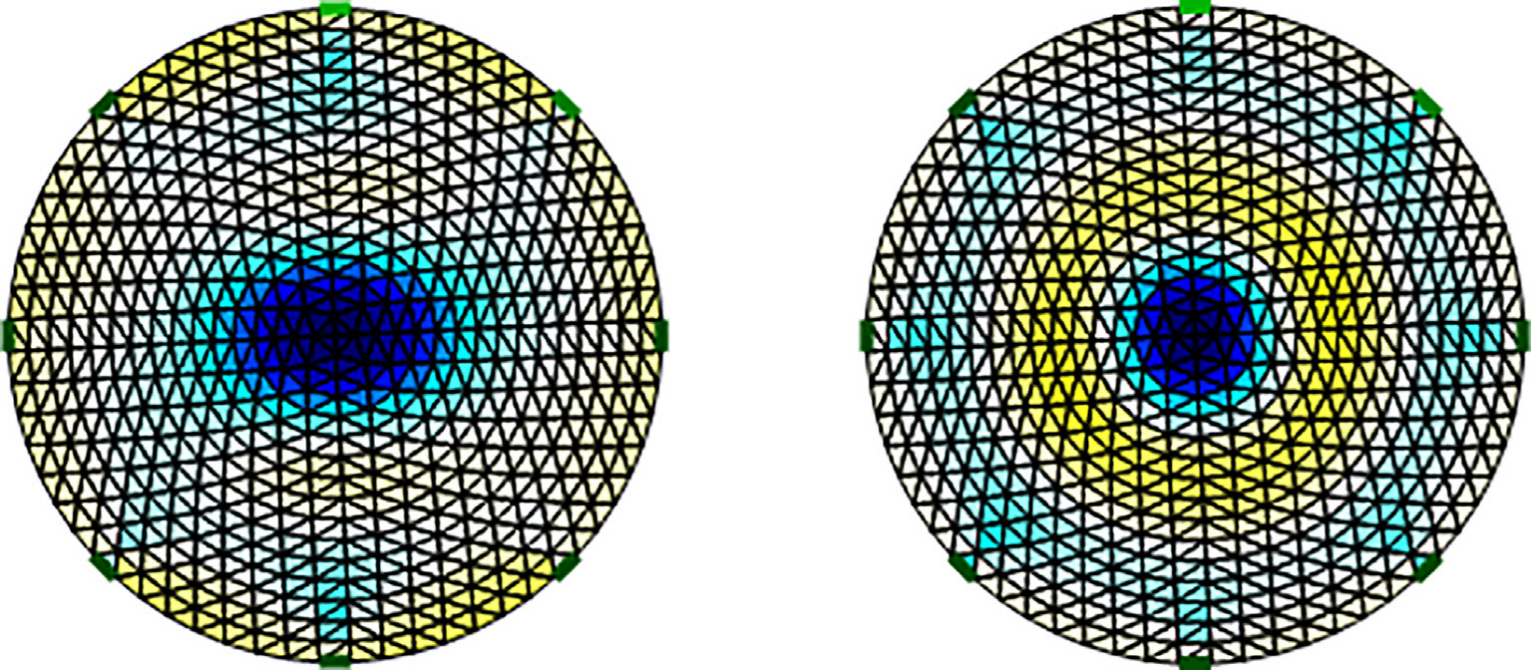
EIT image reconstruction of plastic spheres: small on the left and big on the right.

**Fig. 16:**
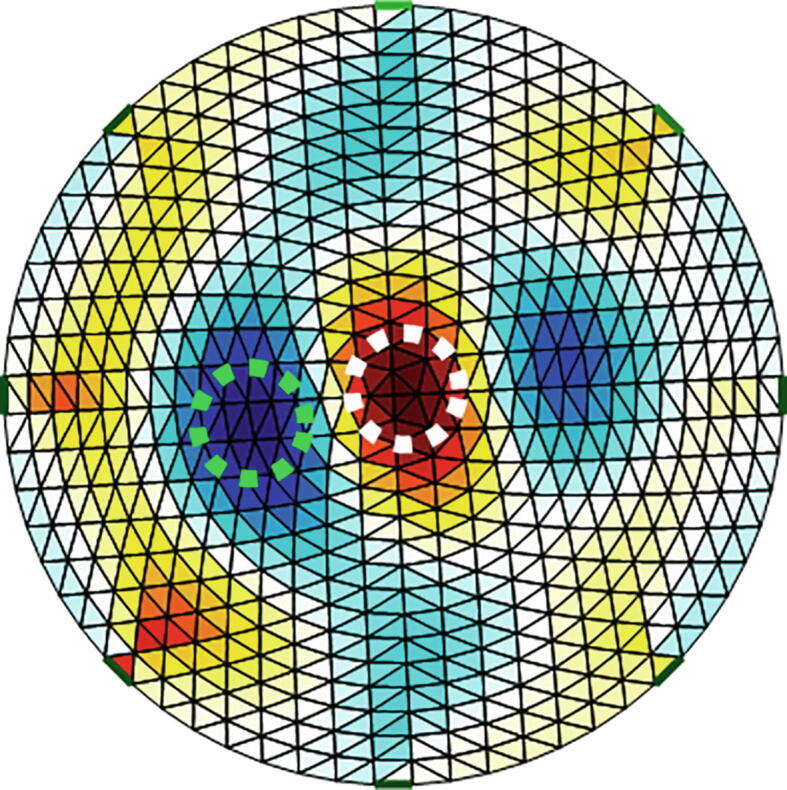
EIT image reconstruction of conductive (dot circle white) and nonconductive (dot circle green) spheres. (For interpretation of the references to colour in this figure legend, the reader is referred to the web version of this article.)

Fig. 14 and Fig. 15 show impedance distributions for the conductive and nonconductive spheres. Both figures show that the proposed system differentiates objects with impedances greater or less than the saline solution. Furthermore, based on impedance distributions, the location of the spheres is estimated. Fig. 16 indicates the impedance distribution when one conductive and nonconductive sphere are simultaneously introduced into the tank. Fig. 14, Fig. 15, Fig. 16 shows the ability of the proposed system to differentiate objects with different conductivity

The conductivity of a stainless-steel sphere is approximately 1.45E6 S/m and that of a plastic sphere is approximately 10E-14 S/m.

## Conclusions

The proposed low-cost BIM system estimates the magnitude and phase of electrical impedances with errors less than 2% and 5%, respectively. The small interquartile ranges presented in Table 3 and Table 4 highlight the low sensitivity to noise of the prototype. Regarding experiments with the saline tank, the Gauss-Newton and Noser algorithms lead to impedance distribution maps where differences in size and conductivity can be easily detected. The prototype is effective to differentiate objects with conductivities ranging from 1E to 13 S/m to 1.45E6 S/m. Furthermore, the BIM system presents the highest SNR (55.23 dB) at 100 frames per second. In this sense, the BIM system is a promising alternative particularly in medical applications where precision and high data acquisition rates are required to determine impedance distributions of human tissues and fluids.

The use of eight electrodes in the proposed BIM system limits the spatial resolution; therefore, future work will focus on the design of BIM systems comprising 16 electrodes with low sensitivity to noise electrodes [39], to improve the estimation of impedance maps. To increase the portability of the prototype, the LabVIEW application will be replaced by another that runs on widescreen Android devices.

## Declaration of Competing Interest

The authors declare that they have no known competing financial interests or personal relationships that could have appeared to influence the work reported in this paper.
